# Real-world outcomes in elderly ALL patients with and without allogeneic hematopoietic stem cell transplantation: a single-center evaluation over 10 years

**DOI:** 10.1007/s00277-022-04793-z

**Published:** 2022-02-19

**Authors:** Kevin D. Hofer, Urs Schanz, Rahel Schwotzer, Gayathri Nair, Markus G. Manz, Corinne C. Widmer

**Affiliations:** 1 Department of Internal Medicine, University Hospital Zurich, University of Zurich, Zurich, Switzerland; 2 Department of Medical Oncology and Hematology, University Hospital Zurich, University of Zurich, Comprehensive Cancer Center Zurich, Raemistrasse 100, 8091 Zurich, Switzerland

**Keywords:** Acute lymphoblastic leukemia, Elderly, Treatment, Allogeneic hematopoietic stem cell transplantation

## Abstract

Elderly patients (EP) of 60 years and above with acute lymphoblastic leukemia (ALL) have a dismal prognosis, but pediatric-inspired chemotherapy and allogeneic stem cell transplantation (allo HCT) are used reluctantly due to limited data and historical reports of high treatment-related mortality in EP. We analyzed 130 adult ALL patients treated at our center between 2009 and 2019, of which 26 were EP (range 60–76 years). Induction with pediatric-inspired protocols was feasible in 65.2% of EP and resulted in complete remission in 86.7% compared to 88.0% in younger patients (YP) of less than 60 years. Early death occurred in 6.7% of EP. Three-year overall survival (OS) for Ph − B-ALL was significantly worse for EP (*n* = 16) than YP (*n* = 64) with 30.0% vs 78.1% (*p* ≤ 0.001). Forty-nine patients received allo HCT including 8 EP, for which improved 3-year OS of 87.5% was observed, whereas EP without allo HCT died after a median of 9.5 months. In Ph + B-ALL, 3-year OS did not differ between EP (60.0%, *n* = 7) and YP (70.8%, *n* = 19). Non-relapse mortality and infection rate were low in EP (14.3% and 12.5%, respectively). Our data indicate that selected EP can be treated effectively and safely with pediatric regimens and might benefit from intensified therapy including allo HCT.

## Introduction

In acute lymphoblastic leukemia (ALL), cure rates as high as 90% are reported in pediatric patients [1]. In contrast, outcomes in adult patients have been found to be substantially worse. With the adoption of pediatric-inspired treatment regimens into adult ALL therapy and a better understanding of the oncogenic landscape with refined risk classification, improved response rates and outcomes especially in Philadelphia chromosome (Ph)-positive ALL have been achieved in recent decades. However, in patients of 60 years and above, outcome is still poor with 5-year overall survival as low as 10 to 20% in patients between 60 and 70 years, and even worse in patients above 70 years [2–5].

The age-related deterioration of outcome is due to several factors. First, high-risk genetic alterations, which can contribute to resistance to conventional chemotherapies, are more frequent with increasing age. Second, treatment-related toxicity of pediatric-inspired chemotherapies with historically reported mortality rates of up to 42% in older patients has led to a conservative use of pediatric-inspired treatments in this patient group [4, 6–11]. Third, the increasing prevalence of comorbidities with age is responsible for the underrepresentation of older patients in clinical trials and the corresponding lack of knowledge about their management. Fourth and lastly, elderly patients (EP) rarely receive allogeneic hematopoietic stem cell transplantation (allo HCT), which continues to be the most effective consolidation therapy for ALL. Historically, allo HCT was restricted to younger patients (YP) because of concerns about high transplant-related mortality (TRM) in patients older than 60 years. However, it could be shown that outcome can be improved in EP with survival rates of 18 to 48% and TRM rates of 21 to 41% when reduced-intensity conditioning (RIC) is used [12–14], which is supported by a recent analysis based on the EBMT registry with 2-year overall survival (OS) of 39 to 53% in transplant-eligible EP above 70 years of age [15].

Overall, these factors result in a substantial bias in treatment knowledge in favor of YP. In the absence of clear treatment guidelines and heterogeneous approaches by different study groups, the management of EP with ALL remains a major challenge in clinical practice. Especially the selection of EP for intensive therapy and allo HCT remains difficult, as withholding intensified treatment from older but fit patients carries the risk of inadequate therapy in a substantial portion of ALL patients. Currently, patients older than 60 years account for around 30% of patients in specialized centers, but this incidence rate is expected to increase due to the aging of society in most developed countries [6, 7].

Here we report real-world outcomes of unselected EP in comparison to YP with ALL with and without allo HCT at our institution over a 10-year period.

## Methods

### Patient population

In this retrospective single-center analysis, data of all patients with a de novo diagnosis of ALL between 2009 and 2019 who underwent treatment at the Department of Medical Oncology and Hematology of the University Hospital Zurich, Switzerland, were analyzed. Burkitt lymphoma/Burkitt cell leukemia were not included in the analysis. Patients younger than 18 years or with a refusal to give general research consent were excluded. Detailed outcome of patients with T-ALL was not studied due to low numbers in EP.

### Definitions

A cut-off of 60 years was applied to separate EP from YP. Central nervous system (CNS) involvement was assessed by evidence of leukemic blasts in the cerebrospinal fluid by morphology and flow cytometry. Lymphadenopathy and spleen size were evaluated with computed tomography. Minimal measurable residual disease (MRD) was determined by PCR of the specific IgH/TCR and/or *BCR-ABL1* level; optimal MRD level was defined as MRD1 < 10^−3^ (after induction) and MRD2 < 10^−4^ (after consolidation). Pediatric-inspired protocols (including a steroid pre-phase, an induction favoring non-myelotoxic drugs such as L-asparaginase, a consolidation with several chemotherapy blocks, and a late intensification) consisted of protocols according to the GRAALL, GRAAPH, or GMALL regimens; non-pediatric protocols were based on hyper-CVAD. Palliative protocols included POMP (purinethol [6-mercaptopurine], oncovin [vincristine sulfate], methotrexate, and prednisone), a single tyrosine kinase inhibitor, or steroids in combination with L-asparaginase. Complete remission (CR) was defined as the presence of less than 5% blasts in the bone marrow and, when available, MRD negativity.

### Allogeneic stem cell transplantation in B-ALL

Patients with high-risk disease, defined as Ph-positive ALL, high white blood cell count (> 30 G/l), CNS involvement, high-risk gene rearrangement (i.e., *KMT2A, IKZF1* deletion), complex (> 5 anomalies), or hypodiploid (< 46 chromosomes) karyotype with available donor were eligible for allo HCT in first CR. Patients with standard risk B-ALL but MRD positivity of ≥ 10^−3^ after induction (MRD1) or MRD ≥ 10^−4^ after consolidation (MRD2) were defined as very high risk and therefore candidates for transplantation in CR1. In addition, allo HCT was recommended to all patients in CR2 after a first relapse. Myeloablative conditioning (MAC) was performed with cyclophosphamide/total body irradiation or busulfan/cyclophosphamide. RIC was used in all EP and consisted of intravenous fludarabine 30 mg/m^2^ (6 days), busulfan 4 × 1 mg/kg body weight per os (2 days, AUC adjusted) and in vivo T-cell depletion with anti-thymocyte globulin (Grafalon®) 10 mg/kg body weight (4 days). For all patients, GVHD prophylaxis was performed with ciclosporin A, which was initially combined with mycophenolate mofetil in patients after RIC and for MAC with a short course of methotrexate.

### Statistical analysis

Descriptive statistics were used for summarizing demographic, disease, and treatment characteristics. To compare nominal variables of baseline characteristics in different subgroups, the chi-square test was used. Student’s *t*-test or Mann–Whitney test was used for continuous variables, depending on data distribution. Overall survival was calculated from the time of diagnosis until death from any cause or last follow-up using the Kaplan–Meier method and differences were compared with the log-rank test. Leukemia-free survival was defined as the time from CR until relapse. A *p* value of < 0.05 was considered significant. Data were compiled using Microsoft Excel (Microsoft Corporation, Redmond, WA, USA), statistical analyses were performed using JMP version 14.0 (SAS Institute, Cary, NC, USA), and survival curves were plotted on Prism GraphPad version 8.4 (GraphPad Software Inc., La Jolla, CA, USA).

## Results

### Patient characteristics

**Fig. 1:**
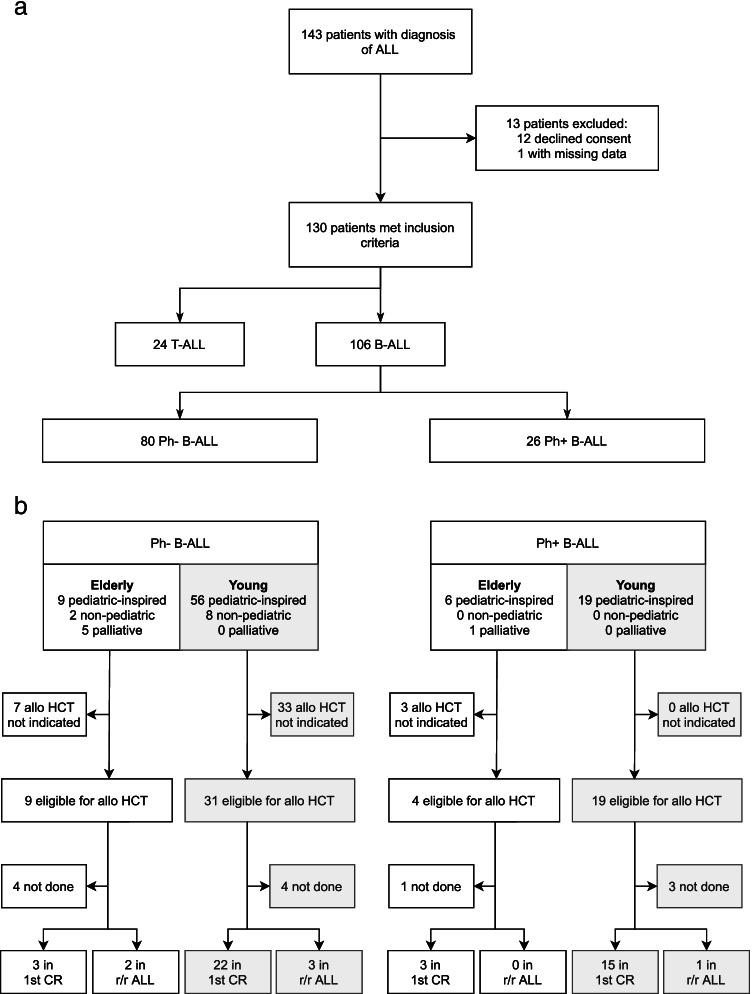
**a** Enrollment of patients. **b** Overview of induction therapy and allogeneic stem cell transplantation based on type of ALL. CR complete response, r/r relapsed or refractory ALL

We identified 143 patients diagnosed with ALL who were treated at our institution. Of these, 13 had to be excluded due to missing data or refusal to give general research consent. A total of 130 patients consisting of 106 B-ALL and 24 T-ALL patients were analyzed (Fig. 1a). Baseline data and disease characteristics of EP and YP are presented in Table 1. Median age was 66 years in EP (range 60–76, *n* = 26) and 39 years in YP (range 18–59, *n* = 104). T-ALL was less common in EP than YP (3/26, 11.5% vs 21/104, 20.2%; *p* = 0.308), whereas incidence of Ph-positive ALL was higher compared to YP (7/26, 26.9% in EP vs 19/104, 18.3% in YP, *p* = 0.374). General risk stratification showed no significant difference between the age groups, but the overall comorbidity rate in EP was high with 84.6% and significantly more cardiovascular (69.2% vs 14.4%, *p* = 0.007) and metabolic (50.0% vs 16.3%, *p* ≤ 0.001) comorbidities as well as prior malignancies (42.3% vs 15.4%, *p* = 0.002) compared to YP. Hemoglobin level, platelet, and white blood cell count as well as blast count did not differ significantly between the age groups. None of the EP in our cohort had CNS involvement at diagnosis, in contrast to 11.5% of YP. Additionally, EP were significantly less likely to have splenomegaly and lymphadenopathy at diagnosis (*p* = 0.001 and *p* = 0.005, respectively).

**Table 1:** Patient characteristics at presentation

	18–59 years *n* = 104	≥ 60 years *n* = 26	*p* value
Median age—years (range)	39 (18–59)	66 (60–76)	–
Female—no. (%)	44 (42.3)	15 (57.7)	0.159
**Immunophenotype**—**no. (%)**			
B-ALL	83 (79.8)	23 (88.5)	0.929
Pro-B	15 (14.4)	6 (23.1)	
Common	40 (38.5)	10 (38.5)	
Pre-B	13 (12.5)	3 (11.5)	
Mature^a^	12 (11.5)	3 (11.5)	
Undefined	5 (4.8)	1 (3.8)	
T-ALL	21 (20.2)	3 (11.5)	0.308
Pro-T	2 (1.9)	1 (3.8)	
Pre-T	6 (5.8)	2 (7.7)	
Cortical	7 (6.7)	0 (0)	
Mature	4 (3.8)	0 (0)	
Undefined	0 (0)	0 (0)	
**ALL subtype**—**no. (%)**			0.374
Ph +	19 (18.3)	7 (26.9)	
Ph −	64 (61.5)	16 (61.5)	
KMT2A-rearranged	8 (7.7)	2 (7.7)	
t(12;21) ETV6-RUNX1	0 (0)	1 (3.8)	
IKZF1 deletion	5 (4.8)	0 (0)	
t(5;14) IGH/IL3	1 (1)	0 (0)	
t(1;19) TCF-PBX1	2 (1.9)	0 (0)	
NOS	38 (36.5)	9 (34.6)	
**Chromosomal abnormalities**—**no. (%)**			0.280
Complex karyotype	8 (7.7)	4 (15.4)	
High hyperdiploidy (51–65 chromosomes)	6 (5.8)	0 (0)	
Hypodiploidy	1 (1)	0 (0)	
Normal karyotype	4 (3.8)	0 (0)	
NA	7 (6.7)	6 (23.1)	
**Risk category (GRAALL protocol)**—**no. (%)**			0.857
Standard risk	66 (63.5)	18 (69.2)	
High risk	10 (9.6)	2 (7.7)	
Very high risk	28 (26.9)	6 (23.1)	
**Comorbidities**—**no. (%)**			
None	48 (46.2)	4 (15.4)	0.004
Cardiovascular	15 (14.4)	18 (69.2)	0.007
Pulmonary	6 (5.8)	4 (15.4)	0.100
Liver disease	0 (0)	0 (0)	–
Gastrointestinal	7 (6.7)	1 (3.8)	0.584
Chronic kidney disease	0 (0)	0 (0)	–
Endocrine and metabolic	17 (16.3)	13 (50)	< 0.001
Neurologic	6 (5.8)	2 (7.7)	0.715
Psychiatric	2 (1.9)	1 (3.8)	0.559
Oncologic	16 (15.4)	11 (42.3)	0.002
Other^b^	20 (19.2)	2 (7.7)	0.190
**Clinical characteristics at diagnosis**^**c**^			
Hemoglobin—g/l (range)	96 (46–169)	99.5 (50–141)	0.812
Platelets—G/l (range)	63 (1–472)	109 (3–761)	0.160
Leucocytes—G/l (range)	0.48 (1.56–95)	8.72 (0.63–196)	0.535
Neutrophils—G/l (range)	1.56 (0.05–32)	1.31 (0.03–68)	0.745
Peripheral blasts—% (range)	40 (0–95)	22 (0–92)	0.208
Bone marrow infiltration—% (range)	90 (23–100)	87 (50–99)	0.160
Splenomegaly—no. (%)	61 (58.7)	6 (23.1)	0.001
Lymphadenopathy—no. (%)	42 (40.4)	3 (11.5)	0.005
Central nervous system involvement—no. (%)	12 (11.5)	0 (0)	0.033

*NA* not available

^a^No Burkitt lymphoma/Burkitt cell leukemia was included in the analysis

^b^Other comorbidities include Turner syndrome, Down syndrome, thalassemia, and osteoporosis

^c^Data given as median with range unless otherwise stated

### Induction therapy and outcome in B-ALL

Generally, it was the primary intention to treat all patients with a pediatric-inspired protocol. In Fig. 1b, the selected treatment regimens, stratified by ALL subtype, as well as the proportion of allo HCT performed in each of the subgroups are outlined. Overall, 83.8% of all ALL patients were treated with a pediatric-inspired protocol. Due to the low number of elderly patients with T-ALL (3/24), this subgroup was excluded from further analysis. In Table 2, detailed results of induction therapy for patients with B-ALL according to age group are listed. In EP, 6 of 23 patients (26.0%) received initial palliative treatment due to comorbidities or reduced Karnofsky performance status. The overall CR rate for EP, regardless of the protocol used, was 73.9% with the highest CR rate (13/15, 86.7%) in patients treated with pediatric-inspired protocols. Information on molecular MRD of B-ALL patients was available in 67 out of 106 (63.2%), including 8 EP. Pediatric-inspired induction therapy resulted in a MRD1 reduction of < 10^−3^ in 2/8 of these EP (25.0%) and in 26/59 in YP (44.1%). MRD2 measurement was obtained in 6 EP, with 50% achieving a MRD < 10^−4^ at this time point. Early death (i.e., within the first 28 days of induction therapy) was low in both age groups treated with pediatric-inspired induction therapy (6.7% in EP vs 2.7% in YP).

**Table 2:** Induction therapy in B-ALL

	**No. of patients**		**CR/CRi**				**MRD1 negative**				**MRD2 negative**				**Early death**^**a**^			
	Young	Elderly	Young		Elderly		Young		Elderly		Young		Elderly		Young		Elderly	
**Protocol**																		
Pediatric-inspired	75	15	88.0%	(66/75)	86.7%	(13/15)	44.6%	(25/56)	25.0%	(2/8)	54.7%	(29/53)	50.0%	(3/6)	2.7%	(2/75)	6.7%	(1/15)
Non-pediatric	8	2	75.0%	(6/8)	50.0%	(1/2)	33.3%	(1/3)	–	–	100.0%	(3/3)	–	–	0.0%	(0/8)	0.0%	(0/2)
Palliative	0	6	–	–	50.0%	(3/6)	–	–	–	–	–	–	–	–	–	–	0.0%	(0/6)
**Overall**	83	23	86.7%	(72/83)	73.9%	(17/23)	44.1%	(26/59)	25.0%	(2/8)	57.1%	(32/56)	50.0%	(3/6)	2.4%	(2/83)	4.3%	(1/23)

^a^Death within 28 days after start of induction therapy

*CR/CRi* complete remission or complete remission with incomplete hematologic recovery

*MRD1* minimal measurable residual disease at around 30 days after the first cycle of induction

*MRD2* minimal measurable residual disease at around 30 days after the second cycle of induction

*OS* overall survival

### Allogeneic stem cell transplantation in B-ALL

Overall, allo HCT was indicated in 63 out of 106 patients with B-ALL (59.5%, Fig. 1b), comparable to the subgroup of EP (13/23, 56.5%). Finally, 8 EP received allo HCT, as 2 were not eligible for transplantation due to comorbidities, and 3 patients refrained from this treatment option (1 with Ph-positive ALL). The main characteristics of patients with B-ALL receiving allo HCT are summarized in Table 3. Results of two YP could not be included in the analysis because allo HCT was performed at another hospital and detailed information was not available. The median age at transplantation in EP was 65 years (range 61–70) and 39 years (range 20–59) in YP. Blinatumomab was used in 8 YP (with subsequent inotuzumab in 1 patient) and 1 EP to achieve CR prior to allo HCT. Patients with Ph-positive ALL as primary indication for allo HCT accounted for 37.5% in EP, which was in the same range as for YP. EP were more likely to receive a graft from a matched unrelated than a mismatched relative donor. All EP underwent RIC, resulting in shorter duration of aplasia (median 5 vs 14 days, *p* ≤ 0.001) and hospitalization (median 29 vs 37 days, *p* = 0.123) compared to YP, who received a myeloablative conditioning including total body irradiation in 78.0%.

**Table 3:** Characteristics of patients with B-ALL undergoing allogeneic stem cell transplantation

Patient characteristics	18–59 years *n* = 41	≥ 60 years *n* = 8	*p* value
Median age—years (range)	40 (20–59)	65 (61–70)	–
Female—no. (%)	21 (51.2)	5 (62.5)	0.559
No comorbidities—no. (%)	23 (56.1)	1 (12.5)	0.024
Karnofsky score—(range)	1 (0.6–1)	0.9 (0.8–1)	0.262
HCT-CI—(range)	5 (0–5)	5.5 (0–8)	0.716*
Months since diagnosis—(range)	5 (2–24)	5.5 (3–35)	0.274
**Disease characteristics**—**no. (%)**			
Immunophenotype			0.936
Ph +	16 (39)	3 (37.5)	
Ph −	25 (61)	5 (62.5)	
ALL status			0.229
CR1	37 (90.2)	6 (75)	
Relapsed/refractory	4 (9.8)	2 (25)	
MRD status			
MRD1 negative	9 (22)	1 (12.5)	0.579
MRD2 negative	9 (22)	2 (25)	0.068
MRD high risk	12 (29.3)	1 (12.5)	0.844
No MRD performed	12 (29.3)	6 (75)	0.014
**Graft**—**no. (%)**			
Donor type			0.407*
Sibling HLA-identical	18 (43.9)	4 (50)	
Matched unrelated	16 (39)	1 (12.5)	
Mismatched unrelated donor	3 (7.3)	1 (12.5)	
Mismatched relative	4 (9.8)	2 (25)	
Stem cell source			0.905*
Bone marrow	10 (24.4)	2 (25)	
Peripheral blood	30 (73.2)	6 (75)	
Cord blood	1 (2.4)	0 (0)	
AB0 incompatibility			0.710*
Identical	25 (61)	4 (50)	
Minor	7 (17.1)	3 (37.5)	
Major	8 (19.5)	1 (12.5)	
Minor and major	1 (2.4)	0 (0)	
CMV status			0.162*
D + R +	14 (34.1)	2 (25)	
D + R −	4 (9.8)	0 (0)	
D − R +	5 (12.2)	1 (12.5)	
D − R −	17 (41.5)	3 (37.5)	
Missing	1 (2.4)	2 (25)	
**Procedure**			
Conditioning regimen—no. (%)			< 0.001
MAC	32 (78)	0 (0)	
RIC	9 (22)	8 (100)	
Total body irradiation—no. (%)	32 (78)	0 (0)	< 0.001
Duration of hospitalization—days (range)	37 (24–92)	29 (26–43)	0.123
Aplasia—days (range)	14 (0–22)	5 (1–12)	< 0.001
Engraftment—days (range)	13 (11–20)	11 (1–17)	0.040

^*^The statistical analysis should be interpreted with caution due to the small numbers involved

*CR1* first complete remission

*HCT-CI* hematopoietic cell transplantation-specific comorbidity index

*MAC* myeloablative conditioning

*MRD* minimal measurable residual disease (MRD high risk defined as MRD1 > 10^−3^ and/or MRD2 > 10^−4^ PCR IgH level)

*RIC* reduced-intensity conditioning

**Fig. 2:**
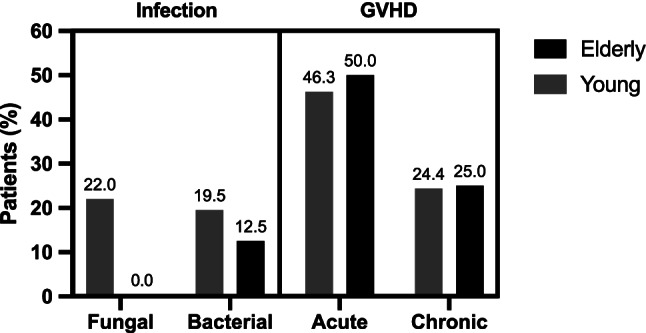
Complications after allogeneic stem cell transplantation. GVHD graft-versus-host-disease

Complications after allo HCT are summarized in Fig. 2. Non-relapse mortality (NRM) was low in both groups with no transplant-associated fatalities in EP and 2/41 deaths (4%) between day 100 and 356 in YP, which were due to graft-versus-host disease and severe infection. No significant difference between EP and YP was observed for acute GVHD (50% vs 46.3%, *p* = 0.661; 25.0% vs 26.8% grade 1, 25.0% vs 12.2% grade 2, 0% grade 3 in both groups, 0% vs 7.3% grade 4) and chronic GVHD (25.0% vs 24.4%, *p* = 0.970). Bacterial infections after allo HCT were rare in EP (12.5%) and no fungal infection was documented in this age group.

### Outcome in B-ALL

**Fig. 3:**
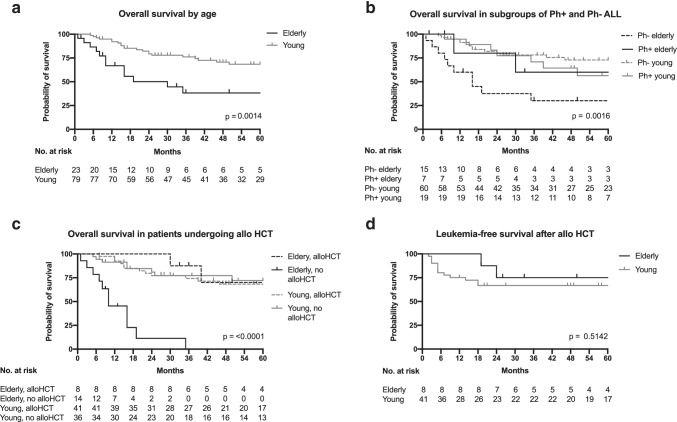
Overall survival and leukemia-free survival in elderly and young patients with ALL based on status of Philadelphia chromosome and performance of allo HCT. Shown are Kaplan–Meier plots for overall survival in elderly and young patients with ALL (**a**), overall survival for subgroups of Philadelphia chromosome-positive and negative ALL (**b**), overall survival in patients undergoing allogeneic stem cell transplantation (**c**), and leukemia-free survival of transplanted patients (**d**). Plots are calculated as the time to death or lost to follow-up, and *p* values calculated by the log-rank test. Tick marks indicate censored data

**Fig. 4:**
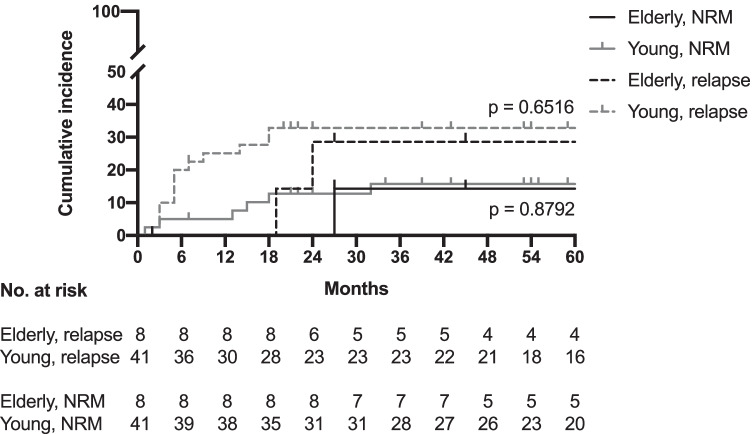
Cumulative incidence of relapse and non-relapse mortality. NRM non-relapse mortality

With a median follow-up period of 16 months (range 6–98) for EP and 39 months (range 1–132) for YP with B-ALL, estimated 3-year OS was significantly different between the two age groups (38.2% [95% CI: 17.1–59.3] in EP and 76.1% [95% CI: 64.3–84.5] in YP, *p* = 0.0014), as shown in Fig. 3a. But when looking at the different ALL subgroups, 3-year OS did not differ significantly between age groups in Ph-positive ALL patients (60.0% [95% CI: 12.6–88.2] vs 70.8% [95% CI: 43.2–86.8], *p* = 0.892), of which only 3/20 received prophylactic post-transplant tyrosine kinase inhibitor therapy. However, in Ph-negative ALL, EP had a markedly reduced survival compared to their younger counterparts (30.0% [95% CI: 9.5–54.0] vs 78.1% [95% CI: 64.6–86.9], *p* ≤ 0.001; Fig. 3b). In YP, OS did not differ if consolidated with or without allo HCT, but numbers were small, so that these results should be interpreted with caution. In contrast, in EP undergoing allo HCT a 3-year OS comparable to OS in YP could be observed (87.5% [95% CI: 38.7–98.1] and 74.3% [95% CI: 57.4–85.3], *p* = 0.912), while EP not eligible for allo HCT were no longer alive after 35 months (median 9.5 months, *p* ≤ 0.001, Fig. 3c). No significant difference in leukemia-free survival after 3 years was found between EP and YP when allo HCT was the consolidation treatment (75% vs 66.7%, *p* = 0.514, Fig. 3d). Cumulative incidence of relapse at 3 years was similar in both groups with 28.6% (95% CI: 0.6–73.3) in EP vs 32.8% (95% CI: 14.6–52.5) in YP (*p* = 0.652). Of note, EP did not relapse before 18 months after allo HCT (Fig. 4). Likewise, no significant difference in NRM could be detected (*p* = 0.880).

Three-year OS in our small T-ALL cohort, which was not studied further, was 74% in YP (*n* = 21) vs 33% in EP (*n* = 3).

## Discussion

In this retrospective study of EP with B-ALL, outcome was generally better than reported results of 5-year OS for patients over 60 years of 10–20% and 3-year OS after allo HCT in first CR between 38 and 43% [4, 8, 16]. However, EP without allo HCT died within less than 35 months after their first diagnosis, of which 62.5% occurred due to disease progression or relapse, while the remaining were due to serious infection or non-infectious multiorgan failure, demonstrating the unchanged dismal outcome in this patient subgroup. Outcome for EP with Ph-negative ALL was particularly unfavorable, whereas EP with Ph-positive ALL had comparable survival as YP, which might be attributable to the good safety profile and high effectiveness of tyrosine kinase inhibitors in relation to cytotoxic chemotherapy in this age group. The 3-year OS for all elderly patients was 38%, with allo HCT increasing the 3-year OS to 87% with a leukemia-free survival of 75%, which was in the range of the outcome for younger transplanted and not transplanted adults. The intensive pediatric-inspired therapeutic regimen could be applied in EP in a high proportion of patients (65%) and was well tolerated with a low early death rate (6.7%) compared to historical rates of up to 35% depending on treatment selection and supportive therapy [17]. The low NRM and favorable outcome after allo HCT in general are most likely due to a combination of different factors such as a consequent supportive therapy including antimicrobial prophylaxis, individual selection of candidates, and consequent use of RIC in EP [18].

The cytogenetic abnormalities were similarly distributed as in populations in other reports [4, 19] except for Ph-like ALL, which could not be assessed, as no data were available for the majority of our patients due to the retrospective nature of the study [20]. However, risk groups were evenly distributed among age groups and did not indicate a relevant disadvantage for EP with the exception of Ph-positive ALL.

Little is known about the exact prevalence and relative contribution of comorbidities in ALL patients and no guidelines exist for the distinction between unfit and fit EP who can tolerate age-adjusted chemotherapy at all. The German Multicenter Study Group for Adult ALL (GMALL) reported a comorbidity rate of 57–92% in patients above 55 years [17] with diabetes, vascular disease, and heart failure accounting for most comorbidities; prior malignancy was present in up to 22%. In our EP cohort, we found a similar comorbidity distribution and rate (84%) but with a higher proportion of prior malignancy (42%). Interestingly, no extra-medullary disease or CNS involvement was documented in our cohort of EP and significantly less splenomegaly or lymphadenopathy was seen than in YP. As younger age is a known risk factor for CNS involvement [21], there are no data published on decreasing likelihood of organ involvement in de novo ALL with increasing age.

The main limitations of this study are its retrospective design and the small patient numbers. However, our findings of excellent outcome after allo HCT in EP are supported by a recent publication of EBMT registry data on elderly ALL patients transplanted in first CR, where a 2-year survival of 50% in patients above 70 years was demonstrated [15]. Also, we are aware that our cohort is subject to some selection bias due to limited referral of unfit highly comorbid EP for treatment assessment to our tertiary center. However, since the majority of bone marrow samples in the catchment area of around 1,500,000 inhabitants are sent for diagnostics to our institution, we assume that almost all patients with a diagnosis of ALL were referred. Additionally, with an estimated incidence of ALL of 1–2/100,000 our patient number of 130 over 10 years lies within a reasonable range and the age distribution with a relative amount of 30% of EP with ALL was similar to results of previous cohort studies [2, 4] as well as the proportion of 25% of Ph + ALL [6, 7]. Furthermore, data on MRD1 and MRD2 were available only in 6/23 (26%) of EP.

In conclusion, we demonstrated that for EP with B-ALL, pediatric-inspired protocols are practicable and allo HCT is a treatment option with low side effects in selected patients at a treatment-experienced center. The appropriate selection of EP for allo HCT remains a challenge and must be made on an individual basis because assessment of organ function precedes therapy and evaluation of how older organs respond to chemotherapy and immunosuppression is difficult to estimate. Previous lifestyle with physical performance in everyday life as well as personal risk tolerance and neurocognitive function should be included in the evaluation. Advanced geriatric assessments with a focus on organ performance in this setting are urgently needed. Further, the role of allo HCT in the era of new effective targeted therapies such as the bispecific T-cell–engaging antibody targeting CD19 (blinatumomab), the antibody–drug conjugate targeting CD22 (inotuzumab-ozogamicin), as well as chimeric antigen receptor–modified T-cells, still needs to be defined and merits further investigation, ideally in the context of clinical trials. But as long as immunotherapies are not available in first line in clinical practice for EP [22], intensive pediatric-inspired protocols and allo HCT should not be deferred a priori based on age, as it can improve outcome in selected patients of 60 years and above.
